# Addressing the challenges of intellectual disability identification for health policy and research in Australia

**DOI:** 10.3389/fpsyt.2025.1703991

**Published:** 2026-01-29

**Authors:** Helen Leonard, Kingsley Wong, Peiwen Liao, Manzoor Khan, Zoe Aitken, Jenny Bourke, Julian N. Trollor, Jenny Downs, Mary-Ann O’Donovan, Anne M. Kavanagh, Preeyaporn Srasuebkul

**Affiliations:** 1 National Centre of Excellence in Intellectual Disability Health, Faculty of Medicine & Health, UNSW Sydney, Sydney, NSW, Australia; 2 The Kids Research Institute Australia, West Perth, WA, Australia; 3 Centre for Health Policy, Melbourne School of Population and Global Health, The University of Melbourne, Melbourne, VIC, Australia; 4 Centre for Disability Studies (CDS), faculty of Medicine and Health, Affiliate of the University of Sydney, Sydney, NSW, Australia

**Keywords:** intellectual disability, definition, consistency, classification, operationalise, data linkage, multisource, health outcomes

## Abstract

This article discusses the important issue of the need for a stable definition of intellectual disability in order to allow comparisons by place and over time such as in the monitoring of this population’s health needs and utilization. The aim of the new Australian National Centre for Intellectual Disability Health, established in 2023, is to ensure that all Australian children and adults with intellectual disability receive high-quality healthcare that meets their needs. Monitoring changes in this regard requires accurate identification of the number of people with intellectual disability within a population which itself is inherently dependent on how intellectual disability is defined. We have used a definition which is relatively easy to operationalize through different sources i.e. a full-scale IQ of less than 70, a condition known to be consistent with intellectual disability or documentation of intellectual disability in medical records; through education a level of intellectual disability defined as either mild/moderate or greater, and through the National Disability Insurance Scheme an International Classification of Diseases (ICD-10-CM) diagnostic code associated with intellectual disability. In contrast the definition required by Inclusion Australia “a lifelong condition that affects a person’s intellectual skills and their behavior in different situations” is much more difficult to operationalize. We discuss this challenge within the context of historical changes and the range of sources possibly able to provide this information today. We present two case studies from different Australian states and conclude with some suggestions for a multi-source approach using data linkage.

## Introduction

Nearly a quarter of a century ago, the critical need for a stable definition of intellectual disability was recognized as essential to enable comparable research across time and place (1). The complexity of defining ‘intellectual disability’ in the face of diverse classification systems and data sources creates a significant challenge for research and policy (2). We acknowledge that prevalence estimates vary across studies due to definitional differences (3), particularly regarding the inclusion in some datasets of borderline intellectual disability functioning (IQ 70-84) which can double the prevalence (4, 5). This challenge persists today and is fundamental to the work and activities of the National Centre of Excellence in Intellectual Disability Health (NCEIDH), establishing the contemporary relevance of the problem. The NCEIDH was established in 2023 with the vision of ensuring that all Australian children and adults with intellectual disability receive high-quality healthcare that meets their needs (6). For NCEIDH to achieve its mission, we must establish a consistent approach to the taxonomy of identifying and defining intellectual disability across our work, particularly given that prevalence estimates vary widely depending on the classification systems used (3, 7). This requires us to be explicit about which definition we employ, the limitations of our data in its alignment with that definition, and to monitor changes in classification systems that might affect our ability to track health outcomes over time.

One purpose of this paper is to argue that for population-level health research and policy monitoring, reliance on any single administrative data source is likely insufficient, and that a validated, multi-source approach may address some of the limitations of single source administrative data. To make this case, the paper is structured as follows. First, we unpack the core challenges in defining and classifying intellectual disability. Second, we present two case studies using state- based data to identify people with intellectual disability. Finally, we discuss the path forward, advocating for a national, collaborative data linkage strategy specifically for intellectual disability. Drawing on recent developments in linked data infrastructure in Australia we highlight the need for a consistent approach to identifying intellectual disability, aligning with a clearly defined definition and outlining the crucial role the NCEIDH can play in leading this work.

## The challenge of defining and classifying intellectual disability

The difficulty in achieving consistent identification stems from both conceptual and practical complexities, as the very construct of ‘intellectual disability’ is multifaceted and has evolved significantly over time. Schalock’s (2011) framework of five critical questions provides a constructive lens through which to understand this evolution (8). The first question, regarding naming, is reflected in the shift from terms like “mental retardation” to “intellectual disability”, though international variations such as “learning disability” in the UK persist (9). The second and third questions, on explaining and defining the construct, highlight the crucial shift from a purely medical or deficit model to a functional, biopsychosocial one. This modern approach, championed by organizations like the American Association on Intellectual and Developmental Disabilities (AAIDD) and articulated in the WHO’s International Classification of Functioning, Disability and Health (ICF), moves beyond pathology to focus on the interaction between a person’s health condition and their environment (10–12). It also distinguishes between an operational definition, necessary for research, and a constitutive definition, which considers a person’s broader life context. The fourth question, on classification, has similarly evolved to prioritize adaptive functioning and support needs over IQ scores alone. Finally, all these elements culminate in the fifth question, concerning public policy, as how we name, define, and classify intellectual disability directly shapes how we understand the health experiences of people with intellectual disability, inform policy decision making, and monitor progress. This conceptual richness, however, is likely to clash with the practical reality of administrative datasets. For a condition with a population prevalence as low as 1-2% we need population datasets with a large sample size. However, such datasets are not designed to capture the nuances of conceptual richness, creating a central challenge which this paper addresses.

The definition of intellectual disability remains complex, with variations in terminology and criteria, many of which are difficult to operationalize, used by clinical, service and advocacy bodies in Australia today. For example, the Australian Commission on Safety and Quality in Health Care uses the DSM-5 definition of intellectual disability as “a neurodevelopmental disorder that begins in childhood and is characterized by intellectual difficulties as well as difficulties in conceptual, social and practical areas of living” (13). On the other hand, Inclusion Australia describes intellectual disability as: “a lifelong condition that affects a person’s intellectual skills and their behavior in different situations” (14). This conceptual diversity creates a fragmented Australian data landscape, where intellectual disability is captured inconsistently across key datasets.

This inconsistency is evident across different data types. National surveys like the Survey of Disability, Ageing and Carers (SDAC) (15) and Household, Income and Labor Dynamics in Australia (HILDA) Survey (16) rely on self- or proxy-reports of “difficult learning or understanding things”, which are hard to align with clinical criteria. Administrative data also has limitations, Centrelink records, for instance, depend on the use of specific payment types and medical codes to determine eligibility (17), while the National Disability Insurance Scheme (NDIS) (18) ‘secondary disability’ field was not previously available to researchers. Clinical data sources are also incomplete (19). Hospital records may provide a diagnostic code, especially where there is a clear medical aetiology but reveal little about a person’s daily functioning. Unfortunately, health claims data (Medicare Benefit Schedule (MBS) and Pharmaceutical Benefit Scheme (PBS)) lack a specific identifier for intellectual disability reducing their applicability to research. A detailed summary of these datasets and their limitations is provided in **Table 1**.

**Table 1:** Summary of information on intellectual disability captured in administrative and relevant survey data.

Domain	Dataset	Survey/admin	Age range	Jurisdiction (state/commonwealth)	Available for linkage	Information	How is intellectual disability defined?^#^	Link to the NDDA?
Disability	Survey of Disability, Ageing and Carers (SDAC)	Survey	All ages	Commonwealth	Linkable through Person Level Integrated Data Asset (PLIDA), please see the paper (21)	Disability information (type & severity), indigenous status, and other demographic data.	Intellectual disability is indirectly identified via self-reported functioning (e.g., ‘difficulty learning/understanding,’; ICF-mappable) and main ICD-coded medical conditions. Prior versions used ‘intellectual.’ Intellectual disability specificity and coding system clarity are limited.	Yes (2021 Census)
	Data Over Multiple Individual Occurrences (DOMINO)	Admin	Children and adults (vary by payment type)	Commonwealth	Yes	Individual live outcomes; wide range of disability characteristics	Intellectual disability is identified via benefit eligibility (payment-dependent); the eligibility determined primarily through medical evidence (e.g., IQ < 70), but also via broader categories from work capacity assessments and carer payment data. ICD codes are broadly categorised (‘intellectual/learning’ and ‘congenital abnormalities’) and limited data are on severity, making identification of intellectual disability less precise.	Yes
	Disability Employment Services (DES)	Admin	Not specified	Commonwealth	No	The data capture claims for people who received services. The information includes disability types and demographics.	The disability diagnosis is ascertained from available certified medical evidence. The primary disability group is reported by employees.	No
	Disability Services National Minimum Data Set (DS NMDS)	Admin	< 65	Commonwealth & State	Yes	Specialist disability support services (under the National Disability Agreement [NDA]). Data includes state, date of birth, sex, Indigenous status, primary disability code, disability groups detailing body structure/function impairments.	It contains data on disability group (primary and other significant). It has ceased functioning in 2018 due to the transition to the NDIS national scheme.	Yes - each state submits minimum data.
	National Disability Insurance Scheme (NDIS)	Admin	< 65	Commonwealth	Yes	Data includes records of support packages for Australians with permanent impairments causing disability.	It provides ICD classification of primary disability and the participant level of function.	Yes
	Justice system	Admin	Adults (≥18)	State	Yes	Statewide Disability Services (SDS) records information regarding offenders with disability (in custody or community).	It has a descriptive indicator for the diagnosis of intellectual disability, but the coding system is uncertain.	Yes - but not the data with disability information.
Hospital	Admission data	Admin	All ages	State*	Yes	It contains reasons for admissions (i.e. diagnoses), procedures and length of stay. Information recorded at the end of a visit.	Intellectual disability is identified via ICD-10-AM codes for: (1) intellectual disability; (2) genetic syndromes inevitably causing intellectual disability.	Yes - each state submits the Admitted Patient minimum data.
	National Hospitals data collection	Admin	All ages	Commonwealth	Yes	It is the major national hospital databases (held by AIHW) including data on Indigenous status (as per sample tables).	Disability status is recorded; however, the details are unclear.	Yes - Part of Admitted patient care (APC) minimum data.
	Emergency Department data collection (EDDC)	Admin	All ages	State	Yes	Includes patient demographics (e.g., age, sex, Indigenous status), emergency department encounter details (e.g., timings, triage, diagnosis, clinical codes), facility information, compensable status, and residence data.	Intellectual disability is identified via general diagnostic codes (ICD-9-CM/-10-AM, SNOMED CT) selected by non-coder staff (keyword/table search); codes are from integrated tables. Specificity for intellectual disability may be limited.	Yes - each state submits the Non-Admitted Patient (NAP) emergency department care minimum data.
	National Non-Admitted Patient Emergency Department Care Database (NNAPEDCD)	Admin	All ages	National and State	Yes	Includes EDDC variables, with additional variables for reporting/linkage: Age (date of birth), Sex/gender, Indigenous status, Remoteness Area (RA), Statistical Local Area (SLA), Statistical Area Level 1 (SA1), Statistical Area Level 2 (SA2), Socio-Economic Indexes for Areas (SEIFA).	It uses the ICD-10-AM (6^th^ to 10^th^ edition), ICD-9-CM (2^nd^ edition), and SNOMED-CT-AU for identification of intellectual disability.	Yes - Part of NAP minimum data.
Out-patient	Mental health	Admin	All ages	State	Yes	Contains clinician-collected data from patient contacts. It includes patient identifiers & demographics (e.g., Project-Specific Person Number [PPN], age, sex, Indigenous status) and service activity records (e.g., visit details, diagnoses, mental health group).	Intellectual disability is identified using coding systems: M (MHCLIC, default), I (ICD-10-ACHI), U (US ICD-10-PCS), C (Canadian ICD-10-CCI).	Yes - each state submits minimum data.
	Non-admitted patient	Admin	All ages	State	Yes	Covers non-admitted patient services with clinical/therapeutic content (requiring medical record notes). Data includes service event details (e.g., dates, outcome) and patient information (e.g., name, age, sex, residence).	Intellectual disability is identified via ICD-9/10, Major Diagnostic Category (MDC), Health Issue Code (HIC).	Yes
	Non-admitted patient	Admin	All ages	Commonwealth	Yes	Non-admitted patient service events from public hospitals/local hospital networks, documenting services (e.g., assessments, consultations, treatments, education) from the patient’s perspective.	This dataset does not contain information on disability.	Yes - each state submits minimum data.
Education	Targeted Specialised Service	Admin	School age	State (NSW)	Maybe	Covers NSW public school students with support needs; includes variables for primary and secondary disabilities.	It provides an indicator for the diagnosis of intellectual disability, but the coding system is uncertain.	Maybe – as part of Nationally Consistent Collection of Data on school students with disability
	Australian Early Development Census (AEDC)	Census	School age - first year	National	Yes	Data is extracted from teacher-completed AEDC Checklist for children in first year of school; updated triennially (participation not mandatory).	Teacher assessment of cognitive skills (school-based) and teacher-reported diagnoses (e.g., Down syndrome, Fragile X, Fetal alcohol spectrum disorder, Autism). Reliability of teacher-reported diagnoses varies.	Yes
	ACT Kindergarten Health Check (ACTKHC)	Health check/questionnaires	School age - first year	State (ACT)	Yes	Data is from ACT Kindergarten Health Check (first-year ACT school children), which covers: vision, hearing, height, weight, development, and parental health history.	Development information from health checks/questionnaires; coding system unclear.	No
Medical services	Medicare Benefits Schedule (MBS)	Admin	All ages	Commonwealth	Yes	MBS covers Medicare-subsidised services (e.g., GP, specialist care). Key data: service dates, Broad type of service, provider information, other relevant details.	No specific diagnostic coding is available for intellectual disability.	Yes
Medicine	Pharmaceutical Benefits Scheme (PBS)	Admin	All ages	Commonwealth	Yes	PBS covers dispensed prescribed medicines under the PBS. Key data: dispensing/prescribing dates, Anatomical Therapeutic Chemical classification system (ATC) codes.	No specific diagnostic coding is available for intellectual disability.	Yes

#This column details if and how intellectual disability is defined or can be ascertained within the dataset, including the coding systems used (e.g., ICD-10-AM, DSM-5), and provides an assessment of its alignment with contemporary understandings of intellectual disability.

*States have similar information on diagnoses and procedures and admission/discharge dates. Private separations in South Australia are not readily available. Western Australia also uses Health Care Financing Administration (HCFA; US) coding in the inpatient data, which specifically refers to Diagnosis Related Groups (DRGs) based on diagnoses and procedures.

Details on diagnostic codes/classifications: the International Classification of Functioning, Disability and Health (ICF) describes disability and health with a focus on people’s functional levels. The International Classification of Diseases (ICD) is a globally used standard diagnostic tool for diseases and health-related activities. Systematized Nomenclature of Medicine – Clinical Terms (SNOMED CT) provides a standardised clinical terminology for defining and conceptualising health information stored in electronic health records; it has been used in ED data in Australia. Mental Health Clinical Intervention Code (MHCLIC) classifies ‘Occasions of Service’ based on clinical information for mental health services. Australian Classification of Health Intervention (ACHI), Canadian Classification of Health Interventions (CCI), and US Procedure Coding System (PCS) are classification systems for health interventions including procedures. Major Diagnostic Categories (MDC) are 25 broad groups that categorise principal diagnoses by body system, and the Health Issue Code (HIC) is used to specify and assign diagnosis codes.

Other abbreviations: New South Wales (NSW), Australian Capital Territory (ACT).

Given this heterogeneity, a clear and consistent definition is required for research purposes. Therefore, this study adopts the definition used by the Western Australian Intellectual Disability (IDEA) Database (20): a recorded IQ score of less than 70 accompanied by evidence of deficits in adaptive functioning. This approach is not proposed as a universal standard, but as a transparent and operationalizable definition which is suitable for analysis within administrative datasets– in line with the criteria put forward by Schalock (8).

While the administrative datasets presented in **Table 1** are individually incomplete, they can potentially be connected through data linkage to build a more holistic picture. Data linkage is the methodological process of connecting records that correspond to the same unique entity across disparate data sources, thereby creating a unified dataset for enhanced statistical analysis. In Australia, this process is often facilitated by a national ‘population spine’, which integrates information from multiple sources such as Medicare, tax, and social security (Centrelink) records to cover almost every resident (21). However, while linkage connects the data, it does not erase the fundamental inconsistencies in how intellectual disability was originally defined and recorded in each dataset. Addressing this challenge should be a key goal of the National Disability Data Asset (NDDA) initiative (22). The NDDA is a new integrated data asset designed to improve outcomes for Australians with disability by bringing together de-identified linked data from sources including government welfare payments and services, the National Disability Insurance Scheme (NDIS), and health services. Datasets are linked at an individual level via a deterministic linkage to a person linkage spine that aims to cover all residents in Australia. Although the NDDA aims to improve disability information, robustly identifying people with an intellectual disability remains a complex issue (2). People with intellectual disability have unique support needs compared to those with other disabilities (23). Therefore accurately differentiating them in national datasets is essential. This requires a sophisticated multi-source approach to defining intellectual disability, a necessity the following case studies will illustrate in practice.

### Example one: the utility of establishing a single dataset for identification of intellectual disability using data from three different sources

To illustrate the practical challenges of identification, we examined Western Australia’s deidentified, population-based intellectual disability database, the Western Australian (WA) Intellectual Disability Exploring Answers (IDEA) database (20). The IDEA Database is a comprehensive, longitudinal data resource currently containing records of individuals with intellectual disability ascertained between 1983 and 2020. It was established through the ongoing linkage of several administrative datasets principally the WA Disability Services Commission (DSC), now the Department of Communities, and the WA Department of Education. More recently, data from the National Disability Insurance Scheme (NDIS) has also been integrated. For context, the NDIS is Australia’s national program for providing individualized disability support to people with a permanent and significant disability (6), Eligibility through DSC required a full-scale IQ of less than 70, a condition known to be consistent with intellectual disability (such as Down syndrome) or documentation of intellectual disability in medical records; through Education a level of intellectual disability defined as either mild/moderate, severe or greater and through NDIS an International Classification of Diseases (ICD-10-CM) diagnostic code associated with intellectual disability (F70-F73, F79) or a condition known to be consistent with intellectual disability (20). Within the NDIS data specifically, individuals can be assigned by the National Disability Insurance Agency (NDA) into disability groups based on “primary disability group” or ICD-based variables. The primary disability groups we used to identify intellectual disability included “Intellectual Disability,” “Developmental delay,” “Global developmental delay,” or “Down syndrome.” Identification using ICD-based variables was based on the following codes: F70 (Mild intellectual disability), F71 (Moderate intellectual disability), F72 (Severe intellectual disability), F73 (Profound intellectual disability), F79 (Unspecified intellectual disability), F84.2 (Rett syndrome), F84.90 (Developmental delay), F84.91 (Global developmental delay), Q87.1 (Cornelia de Lange syndrome), Q87.8 (Coffin-Lowry syndrome), Q90 (Down syndrome), Q91 (Edwards syndrome and Patau syndrome), Q93.4 (Cri du Chat syndrome), Q93.5 (Angelman syndrome), and Q99.2 (Fragile X syndrome). As participants can have up to six secondary disabilities recorded, the presence of intellectual disability in any of these fields was also considered sufficient for positive identification. Data from any or all ascertainment sources was used to determine eligibility. The ethical oversight of the IDEA Database is managed under HREC 2014/24 - Intellectual Disability Exploring Answers (IDEA) Database: Infrastructure Linkage.

We assessed the sensitivity of the WA NDIS intellectual disability indicators, using the IDEA database as the gold standard. Sensitivity referred to the proportion of individuals in the IDEA reference database who were also identified in the NDIS data. To maximize identification, we created derived indicators combining primary disability groups, primary ICD-coded diagnoses, and secondary disability codes. The specific terms and diagnostic codes used for this identification are detailed in the notes for **Table 2**.

**Table 2:** Sensitivity of different NDIS variable combinations in identifying intellectual disability in Western Australia.

Sensitivity					
IDEA diagnosis category	Primary disability [Group]	Primary disability [ICD]	Primary disability [Group] + primary disability [ICD]	Primary disability [ICD] + secondary disability	Primary disability [Group] + primary disability [ICD] + secondary disability
ID with or without ASD	43.6%	38.4%	43.8%	50.8%	55.1%
ID with ASD	9.4%	8.5%	9.5%	22.4%	23.2%
ID without ASD	91.0%	80.0%	91.5%	91.2%	99.3%
ASD not ID	1.0%	0.8%	1.0%	1.5%	1.7%

ID, intellectual disability; ASD, autism spectrum disorder.

We acknowledge that the terminology used for autism and autism spectrum disorder varies internationally and also in the way that people like to be described. We have used the terminology generally accepted in Australia and apologise for this.

An individual was determined to have intellectual disability when their primary disability group included any of the following terms: “Intellectual Disability,” “Developmental delay,” “Global developmental delay,” or “Down Syndrome.”

For the ICD-based variables, identification was based on the following codes: F70 (Mild intellectual disability), F71 (Moderate intellectual disability), F72 (Severe intellectual disability), F73 (Profound intellectual disability), F79 (Unspecified intellectual disability), F84.2 (Rett syndrome), F84.90 (Developmental delay), F84.91 (Global developmental delay), Q87.1 (Cornelia de Lange syndrome), Q87.8 (Coffin-Lowry syndrome), Q90 (Down syndrome), Q91 (Edwards syndrome and Patau syndrome), Q93.4 (Cri du Chat syndrome), Q93.5 (Angelman syndrome), and Q99.2 (Fragile X syndrome). Since participants could have up to six secondary disabilities, the presence of intellectual disability in any of these variables was also considered sufficient for a positive diagnosis.

The analysis revealed significant variation in the NDIS dataset utility based on co-occurring conditions. As expected, the sensitivity for identifying individuals with intellectual disability from all categories was highest when all diagnostic information was utilized. Overall sensitivity of identifying individuals with intellectual disability was 55.1%. However, our findings reveal a striking disparity; while the NDIS demonstrated very high sensitivity (99.3%) for identifying individuals with intellectual disability alone, sensitivity dropped dramatically to 23.2% for individuals with co-occurring intellectual disability and autism spectrum disorder. This 76.1 percentage point difference (95% confidence interval 75.2,76.9; z-test P<0.001) highlighted a critical gap in the ability of the NDIS to capture the full spectrum of intellectual disability, particularly affecting those with complex diagnostic profiles.

Beyond autism comorbidity, other factors also influenced identification (**Table 3**). For example, individuals with mild or moderate levels of intellectual disability, males, younger cohorts, those ascertained solely through the public education department and those who were born outside WA were less likely to be identified in the NDIS dataset. These patterns demonstrate that relying on a single data source such as the NDIS can create a biased and incomplete evidence base. If the true population is not captured in a dataset, there are major implications for research and the development of policies and evaluation of their impacts and equities.

**Table 3:** Characteristics of Western Australian individuals with intellectual disability (with or without ASD), by NDIS identification status.

Characteristic	NDIA identification status		*p*-value
	Identified (N = 9,244) 55.0%	Unidentified (N = 7,549) 45.0%	
Sex			
Female	3,759 (69.8)	1,627 (30.2)	<0.001
Male	5,467 (48.2)	5,878 (51.8)	
Unknown	18 (29.0)	44 (71.0)	
Year of birth			
1940-1989	3,349 (92.6)	268 (7.4)	<0.001
1990-1996	1,312 (81.5)	297 (18.5)	
1997-2002	1,618 (65.5)	853 (34.5)	
2003-2008	1,533 (38.4)	2,464 (61.6)	
2009-2016	1,432 (28.1)	3,667 (71.9)	
Ascertainment source			
DSC	2,986 (85.8)	495 (14.2)	<0.001
EDU	664 (15.7)	3,563 (84.3)	
DSC & EDU	4,566 (56.8)	3,472 (43.2)	
NDIA	1,028 (98.2)	19 (1.8)	
WA born			
Yes	3,406 (72.5)	1,293 (27.5)	<0.001
No	5,838 (48.3)	6,256 (51.7)	
Indigenous status			
Non-indigenous	8,145 (53.8)	6,992 (46.2)	<0.001
Indigenous	877 (70.6)	366 (29.4)	
Unknown	222 (53.8)	191 (46.2)	
IDEA intellectual disability level			
Mild	3,754 (44.9)	4,604 (55.1)	<0.001
Mild or moderate	538 (31.5)	1,168 (68.5)	
Moderate	3,712 (69.5)	1,626 (30.5)	
Severe	1,240 (89.1)	151 (10.9)	

DSC, Disability Services Commission; EDU, Department of Education; NDIA, National Disability Insurance Agency.

* Definitions of the level of intellectual disability approximate to IQ or adaptive functioning scores of mild (IQ 55-69), mild or moderate (IQ 40-69), moderate (IQ 40-54) and severe (IQ<40). p-values are derived from chi-squared test of independence.

### Example two: The multi-source approach in New South Wales

This illustrates a multi-source, bottom-up approach used in NSW to identify people with intellectual disability from existing administrative data (24, 25). The method is detailed in a cohort profile of over 100,000 individuals who accessed disability and health services in NSW between 2001 and 2018. In contrast to the WA case study which builds from core disability data. the NSW model combined multiple, individual health and disability records within a linked data infrastructure, creating a rich longitudinal resource (25).

Individuals were included in the NSW intellectual disability cohort if any of their records contained a diagnosis or service related to intellectual disability. The datasets used for identification were extensive, including the Disability Services Minimum Data Set (DS-MDS); health services records from the Admitted Patient Data Collection (APDC) and the Emergency Department Data Collection (EDDC) and Mental Health Ambulatory Data Collection (MH-AMB); data from targeted specialist support services in public schools; and records from the NSW Ombudsman, the NSW Public Guardian, and State-wide Disability Services for offenders in custody (25). While standard diagnostic criteria are generally applied, some variation existed across these sources, potentially including a small number of individuals with borderline intellectual functioning. This reflects real-world service delivery patterns and strengthens the case for multi-source validation.

The linkage performed by the NSW Centre for Health Record Linkage (CHeReL) (26) and the AIHW Data Linkage Unit (27), creates an invaluable resource for examining health outcomes in this population. While this model provides a robust pathway for ascertainment, validation against methods is crucial. For instance, the cross-sectional prevalence of intellectual disability identified by the NSW cohort was 1.1% (24) which is lower than the birth prevalence of 1.7% estimated from the WA IDEA Database for a similar time period (28). The NSW method may be more efficient at identifying adults who interact with services, whereas the WA method, with its inclusion of education records, is stronger at capturing childhood prevalence. The lower prevalence in the NSW adult cohort may be partially explained by the “transition cliff” (2) – poorer ascertainment in adulthood – and the fact that birth prevalence does not account for mortality over the life course.

### The path forward: a collaborative, multi-source solution

The case studies from WA and NSW (20, 25) confirm a fundamental principle: integrating multiple data sources is an ideal way to build a complete picture of a population. This approach moves beyond a siloed service-centric view to a more holistic one – a strategy already proven effective at a state level (25).

Australia is now well-positioned to apply this principle nationally through its major data linkage infrastructure. This includes initiatives such as the National Health Data Hub (NHDH) (29), which aims to streamline access to linked health data, the disability-focused National Disability Data Asset (NDDA) (22), as well as the Person-Level Integrated Data Asset (PLIDA) platform (30). Together, these initiatives aim to integrate health, disability, and social service data to address complex questions. In relation to intellectual disability, these national linked data assets will be transformative for generating evidence to better understand the health of people with intellectual disability. Intellectual disability indicators created from multiple sources of data have the potential to identify a large population of Australians with intellectual disability as has been done to generate an identifier for Indigenous status (31). However, the indicators in the first instance may not be representative and ongoing efforts will be needed to improve the quality of data, address known data gaps (underrepresented populations) through inclusion of additional datasets, and generation of statistical methods to ensure representativeness. For example, the inclusion of primary care data such as GP records (32), which are not currently linked to any of the existing assets, are likely to improve the representativeness of the intellectual disability indicators.

This collaborative approach is already being actioned. The NCEIDH, as part of its work program, is providing scientific leadership by developing and validating a national algorithm to identify people with intellectual disability using these linked assets. This work is crucial for creating a more accurate national research and policy tool, and the future validation of this algorithm against state-based registers, such as the WA IDEA Database, also remains valuable if future linkage opportunities permit. To realize the full potential of our national data, a coordinated effort is needed. Data custodians, researchers, clinicians and government agencies must continue to work together to support data linkage, improve the accessibility of key data fields for research and ensure findings are fed back to improve services (not only health but also intersecting systems such as early intervention, rehabilitation, social care, and education) for all Australians with intellectual disability.

## Conclusion

The challenge of identifying individuals with intellectual disability consistently and reliably across Australia persists, nearly a quarter of a century after its critical importance was first highlighted (1). This manuscript underscores that without robust national data, the ability to monitor health outcomes, plan services, and develop informed policy is significantly hampered. Our findings, particularly the limitations of single-source data, demonstrate an urgent need for the refined, multi-source approaches outlined. Ultimately, the goal is to foster a data landscape that provides a much clearer, more accurate understanding of the prevalence, needs, and health outcomes of Australians with intellectual disability. Such an evidence base is fundamental for the NCEIDH and all stakeholders to effectively fulfil their missions, ensuring that policies and services lead to tangible improvements in health equity and quality of life.

## Data Availability

The datasets presented in this article are not readily available because of ethical restriction. Requests to access the datasets should be directed to HL, helen.leonard@thekids.org.au.
